# SBRT Treatment of Multiple Recurrent Auricular Squamous Cell Carcinomas Following Surgical and Conventional Radiation Treatment Failure

**DOI:** 10.7759/cureus.325

**Published:** 2015-09-17

**Authors:** Drew Brotherston, Ian Poon

**Affiliations:** 1 Radiation Oncology, Odette Cancer Centre, Sunnybrook Health Sciences Centre

**Keywords:** stereotactic body radiotherapy, non melanoma skin cancer, re-irradiation, cancer recurrence, squamous cell carcinoma

## Abstract

The treatment of recurrent skin cancers of the head and neck following curative doses of radiotherapy and/or surgery is usually palliative radiation therapy (RT) but with mediocre control rates leading to symptomatic local recurrences. We present a 93-year-old male treated with 50 Gy in five fractions for a subauricular cutaneous squamous cell carcinoma who initially underwent partial auriculectomy and accelerated concomitant boost radiotherapy (60 Gy in 21 fractions over 23 days), and then two additional surgeries ending with completion auriculectomy. Re-irradiation with SBRT was well tolerated despite prior high-dose therapy.

## Introduction

Non-melanoma skin cancers (NMSCs) are often managed using definitive surgical excision. In patients where surgical management is deemed not to be appropriate, radiotherapy may be considered for both definitive and palliative therapy [1-2]. Studies of radiation therapy (RT) as definitive therapy in primary and recurrent epithelial skin cancer consistently report high rates of local control despite extremely variable total dose and fractionation schedules [3-7]. 

Studies using radiotherapy in the management of NMSC use field-based RT regardless of fractional dose or regimen (e.g. hypo or hyperfractionated schedules). There is little data reported in the literature regarding re-irradiation of non-melanoma skin tumours with modern advanced techniques.

In clinical practice, cases of recurrent NMSC following curative surgery/RT or where definitive therapy is contraindicated, treatment is typically palliative RT. Reporting in the literature is limited, but a small cohort study within our centre found palliative RT provided only moderate local and symptomatic control (58% and 61%, respectively, for 28 patients) [8].

Stereotactic body radiotherapy (SBRT) is able to deliver high-dose radiation precisely while sparing normal adjacent tissues and has demonstrated promise in the treatment of many disease types where typical surgical, radiation, and or chemotherapy regiments are not feasible [9-14]. No reports exist of SBRT use in the treatment of cutaneous lesions; however, hypofractionated regimens using conventional RT have showed good rates of local control with little toxicity when used as definitive therapy or cases of salvage [5-6].

We present a case of a 93-year-old male with multiple recurrent auricular epithelial squamous cell carcinoma (SCC) involving the left ear treated with three surgeries, including an initial surgery that required adjuvant RT for positive margins with regrowth on radiation that was eventually successfully controlled with SBRT.

Informed patient consent was obtained for treatment and publication. Details that might disclose the identity of the subject under study were omitted.

## Case presentation

**Figure 1:**
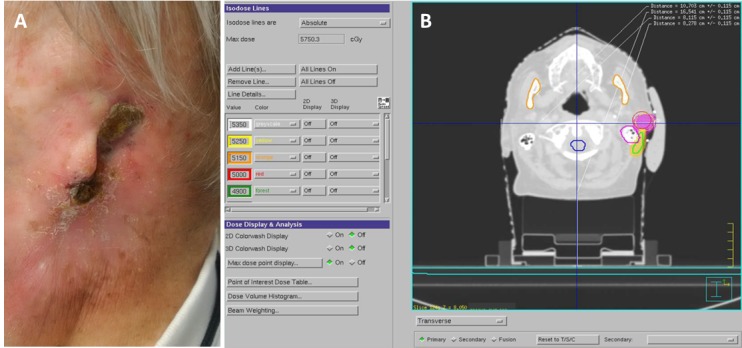
Stereotactic Treatment of Recurrent Lesion A) Left auricle with recurrent SCC. B) Radiation treatment plan for recurrent lesion.

Initially, in late October 2013, a 93-year-old male presented with a six-month history of pain and swelling of the left ear. Clinical examination demonstrated a lesion along the posterior auricular sulcus with exposed cartilage within the depth of the lesion. Extending beyond this lesion was a region of erythema that suggested early involvement. There was no evidence of regional metastatic spread. A previous biopsy confirmed the lesion to be a poorly differentiated squamous cell carcinoma. The lesion was treated by surgical resection via a partial auriculectomy removing a 3 cm tumour. The deep surgical margin was positive, necessitating adjuvant radiation therapy. This was initially prescribed by the referring radiation oncologist with a hypofractionated regimen of 45 Gy in 15 fractions delivered daily using a single lateral electron field – 15 MeV with bolus. This hypofractionated lower dose regimen was originally pursued due to the advanced age of the patient. After the third fraction, the lesion had regrown to 3 cm, prompting an additional 15 Gy in six fractions as a concomitant boost for a total of 60 Gy in 21 fractions delivered over just 23 days, completed in January 2014. In April 2014, an irregular skin lining was noted within the treatment site, which was confirmed to be disease recurrence after a biopsy. A near-total auriculectomy sparing the lobe was performed, with final pathology showing a moderately differentiated squamous cell carcinoma and negative margins. Six months later in October 2014, a new squamous cell carcinoma was noted on the lobule of the remaining pinna, and a third completion auriculectomy was performed. Pathology revealed a poorly differentiated squamous cell carcinoma with negative margins. Three months later, in January 2015, a recurrent mass developed at the base of the left auricle extending into the previous treatment site and the external ear canal with an invasion of the parotid gland (Figure 1A). The gross tumour volume was treated with 50 Gy in five fractions twice a week in 2.5 weeks. A high dose clinical target volume was not added and only a 2 mm circumferential expansion to the gross tumour volume was used for the final planning target volume. The suspicious erythematous area surrounding the gross tumor volume (GTV) received 40 Gy in five fractions concurrently as part of the same plan (Figure 1B). Within one month post-treatment, the tumour had completely regressed with no residual disease. No significant complications, such as bleeding or persistent or high-grade dermatitis, bone, or cartilaginous necrosis were noted.

## Discussion

The successful control of a skin SCC with multiple recurrences is unusual. There are several studies in the literature that report the use of definitive radiotherapy with hypofractionated schedules in the treatment of recurrent NMSC [3-7]; however, these are exclusive following failure or positive margin status after surgical excision. Definitive treatment of previously irradiated tissue in NMSC has rarely been reported and only in small samples [15-16]. Indeed, with SCCs of the skin that are refractory to local therapies, the approach typically shifts to systemic therapy, although patients are frequently not considered good candidates due to advanced frailty [17]. Furthermore, to the knowledge of the authors, no study has described using SBRT in recurrent (or even primary) NMSC.

In general, the use of repeat radiation therapy in the treatment of cutaneous skin cancers is not used and reports in the literature are scant. This is perhaps due to fears of severe toxicities (bleeding, ulceration, tissue necrosis) that have been demonstrated during re-irradiation using conventional RT techniques in mucosal tumours [18-21], which require the addition of large normal tissue margins. One of the primary benefits of SBRT is a steep dose drop-off, which allows for precise dose delivery to target volumes while sparing normal tissue, thereby increasing radiation treatment effectiveness and avoiding significant toxicity. This case demonstrates the power of this approach for, despite the SBRT volume being within the previously irradiated tissue and a triple surgical site, no significant toxicity (such as ulceration, bleeding, or tissue necrosis of epithelium, cartilage, or bone) was observed.

This case is perhaps most unique due to the ability of SBRT to achieve a complete response in an extremely resistant lesion. Over the course of 17 months, the disease-free interval of the lesion did not exceed six months. Following adjuvant RT, the time to failure was just 3.5 months with postoperative regrowth of the lesion at fraction three during this radiation course. In an article by Brown, et al., the authors suggest that large doses delivered over a short duration may lead to an additive cell kill that is different from conventional RT [22]. This could potentially explain the differing control outcomes of the two schedules used in this case. However, even when using a traditional biological equivalent dose, the original radiation dose (assuming α/β ratio of 10 for early responding tissues) was 77 Gy versus 100 Gy for the SBRT regimen. This was part of the motivation for choosing such a high dose per fraction as schedules of less than 10 Gy per fraction would not have delivered a biologic dose that was substantially greater than the original therapy, which failed to control the tumour. In a sense, this case allows for direct comparison of conventional RT versus SBRT where, despite a reduced total dose, the hypofractionated approach has rendered a more complete and sustained response.

With good rates of response and low high-grade toxicity, SBRT should perhaps be considered early in the treatment of refractory cutaneous SCCs. This case demonstrates, at least with a small volume, that an extremely high radiation dose can be tolerated well and result in a good response. In a study of over 400 NMSC cases treated using various hypofractionated schedules, van Hezewijk, et al. [6] concluded that the T stage is the only significant factor for recurrence. This potentially implies that the dose may have to be adapted to the treatment volume to achieve control for larger tumours. However, with larger treatment volumes, there is a greater potential for toxicity. Therefore, further studies are needed to determine whether SBRT can be applied broadly in the management of refractory SCCs or if it is best reserved for a subset of patients with lesser disease burden as in this case.

## Conclusions

We describe a rare scenario of a patient with multiple recurrent subauricular SCC treated with SBRT after recurrence following partial auriculectomy, plus accelerated concomitant boost RT and repeat surgical failure (near total auriculectomy followed by complete auriculectomy), who achieved a complete response of the primary lesion with no significant toxicity.
